# Metal Levels in Delaware Bay Horseshoe Crab Eggs from the Surface Reflect Metals in Egg Clutches Laid beneath the Sand

**DOI:** 10.3390/toxics11070614

**Published:** 2023-07-14

**Authors:** Joanna Burger

**Affiliations:** 1 Division of Life Sciences, Rutgers University, 604 Allison Road, Piscataway, NJ 08854, USA; burger@dls.rutgers.edu; 2 Environmental and Occupational Health Sciences Institute, Rutgers University, 170 Frelinghuysen Road, Piscataway, NJ 08854, USA

**Keywords:** Cd, Pb, Hg, Se, food web, shorebirds, keystone species

## Abstract

Understanding variations in metal levels in biota geographically and under different environmental conditions is essential to determining risk to organisms themselves and to their predators. It is often difficult to determine food chain relationships because predators may eat several different prey types. Horseshoe crab (*Limulus polyphemus*) eggs form the basis for a complex food web in Delaware Bay, New Jersey, USA. Female horseshoe crabs lay thumb-sized clutches of eggs, several cm below the surface, and often dislodge previously laid eggs that are brought to the surface by wave action, where they are accessible and critical food for migrant shorebirds. This paper compares metal and metalloid (chromium [Cr], cadmium [Cd], lead [Pb], mercury [Hg], arsenic [As] and selenium [Se]) concentrations in horseshoe crab eggs collected on the surface with concentrations in eggs from clutches excavated from below the sand surface, as well as examining metals in eggs from different parts of the Bay. The eggs were all collected in May 2019, corresponding to the presence of the four main species of shorebirds migrating through Delaware Bay. These migrating birds eat almost entirely horseshoe crab eggs during their stopover in Delaware Bay, and there are differences in the levels of metals in blood of different shorebirds. These differences could be due to whether they have access to egg clutches below sand (ruddy turnstones, *Arenaria interpres*) or only to eggs on the surface (the threatened red knot [*Calidris canutus rufa*] and other species of shorebirds). Correlations between metals in clutches were also examined. Except for As and Cd, there were no significant differences between the metals in crab egg clutches and eggs on the surface that shorebirds, gulls, and other predators eat. There were significant locational differences in metal levels in horseshoe crab eggs (except for Pb), with most metals being highest in the sites on the lower portion of Delaware Bay. Most metals in crab eggs have declined since studies were conducted in the mid-1990s but were similar to levels in horseshoe crab eggs in 2012. The data continue to provide important monitoring and assessment information for a keystone species in an ecosystem that supports many species, including threatened and declining shorebird species during spring migration.

## 1. Introduction

Global changes in coastal habitats, including increasing human populations, increasing temperatures, sea level rise, loss of coastal habitat, and the spread of contaminants have captured the attention of the public. These threats have resulted in a global loss of biodiversity [1,2,3,4], including in coastal and estuarine habitats [5,6]. The public, health officials, conservationists, and governmental agencies are also interested in levels of contaminants in biota that directly and indirectly affect species within their environment, and that ultimately can affect people through the food web [7,8,9,10,11,12]. Concern for wildlife exposed to these threats has forced government agencies to address contaminants in the environment, often with regulations [13,14]. However, regulations, whether to protect species or to reduce contaminants, require basic assessment of chemicals in a range of species on the food web. Particularly important, however, are species that are either at the base of the food chain or at the top of the food chain [7,14,15,16,17]. Species at the top of the food chain are the species usually examined [18], largely because they bioaccumulate toxic chemicals, making them easier to quantify. Examining species at the bottom of the food web provides information on potential exposure to their predators [18,19]. It is useful to select a species that is eaten by several different species in the food web, providing maximum information.

This paper examines metals in the eggs of Atlantic horseshoe crabs (*Limulus polyphemus*) in the springtime, when they are spawning along the beaches of Delaware Bay. Objectives were to (1) examine levels of several metals in eggs at the tide line or in the surf (called surface eggs), (2) compare the metals in eggs from the surface with intact clutches laid several cm below the sand [20,21], (3) examine locational differences in metals in the Bay, (4) determine whether there are correlations between metals in the clutches, and (5) compare current levels to previous levels. Each clutch is laid by one female digging below the sand surface. This often disrupts previously laid clutches, releasing eggs that are then carried to the surface. The first objective is important because it is these surface eggs washed up by the surf that are available for consumption by shorebirds, young turtles, and small fish [18,20,21,22]. During the spring migration stopover in Delaware Bay, shorebirds of several species depend almost entirely on the eggs of the crabs to store fat for long non-stop flights north to breeding grounds [23]. The second is important because when females lay clutches in the sand, the clutches are held together by an exudate the female lays with the eggs—this exudate may contain more or less contaminants than the surface eggs. The third objective is important because locational difference implies that their predators are exposed differentially on different beaches. The fourth is important because it serves as an indicator of the metals of females that laid each clutch. Finally, understanding contaminants in horseshoe crab eggs is important as one indicator of the health of the estuarine ecosystem.

The Atlantic horseshoe crab (hereafter called horseshoe crab) occurs from Maine to Florida and along the Gulf coast, although populations are concentrated off the coast of the New York Bight [24]. Horseshoe crabs are harvested for bait, and their harvest is controlled so that there are sufficient eggs not only to maintain crab populations but provide excess eggs for the shorebirds during migration [25,26]. The crabs spend the winter out on the continental shelf, and each spring migrate into bays and estuaries to spawn on the beaches [27,28]. When populations of the red knot (*Calidris canutus rufa*) began to decline precipitously in the 1990s, the importance of the eggs of horseshoe crabs to migrating shorebirds in Delaware Bay was established [29,30]. Red knots and other shorebirds migrating through Delaware have only 2–3 weeks to eat enough crab eggs to nearly double their weight with fat to fuel their migration north to Arctic breeding grounds [31,32,33,34]. Extensive, multi-disciplinary studies have shown the clear relationship between abundance of horseshoe crab surface eggs, shorebird time on Delaware Bay, weight gain, and success on the breeding grounds [22,31,35,36,37,38,39].

The populations of horseshoe crab are currently managed by the Atlantic State Marine Fisheries Commission (ASMFC), guided by a technical committee [24,25]. The technical committee was established when it was clear that there was a relationship between decline in horseshoe crab eggs on Delaware Bay beaches, and shorebird declines (particularly of red knots [29,30,31,32,33]). The ASMFC [25] approved and released the first horseshoe crab management plan that considered not only the stock needed to continue to maintain horseshoe crabs, but numbers that were sufficient to produce enough eggs for shorebirds. It was the first fishery management plan that considered two species (the crabs and shorebirds). Shorebirds are one of the most endangered group of birds in the world, and the four main shorebird species migrating through Delaware Bay in spring have the greatest declines of shorebirds overall [40,41,42]. Since the primary food of red knots and other shorebirds while on Delaware Bay is horseshoe crab eggs, understanding how metal levels vary is important. Levels of metals in horseshoe crab eggs and muscles vary by location on Delaware Bay beaches and by year for some metals [43,44,45,46,47], As well as in early life stages of eggs and larvae [48].

## 2. Material and Methods

**Figure 1:**
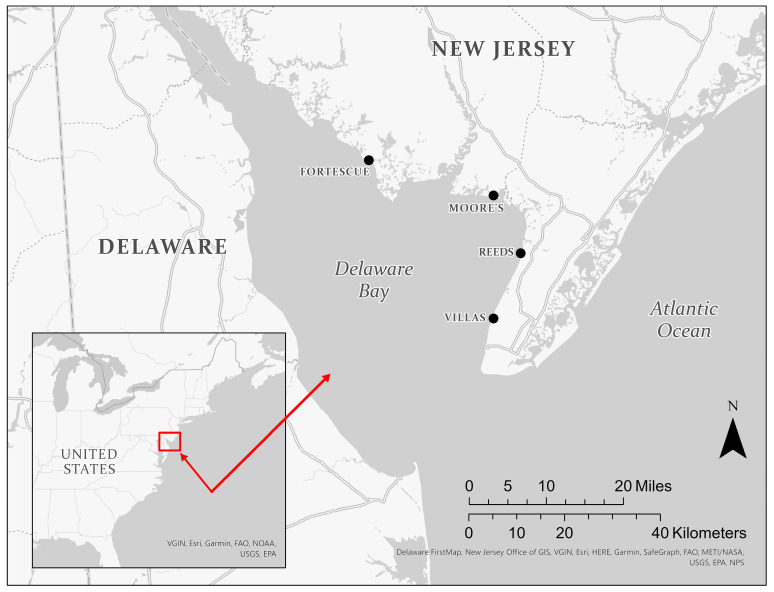
Map of Delaware Bay (New Jersey) showing the locations where horseshoe crab eggs were collected in May 2019.

The overall protocol was to collect eggs of horseshoe crabs so that comparisons could be made between the metals in eggs on the surface and in clutches (from Reeds Beach), and from clutches at four different sites (from south to north: Villas, Reeds, Moore’s, Fortescue) (Figure 1). All eggs were collected on the New Jersey side of Delaware Bay in May 2019.

### 2.1. Field Collection

Egg clutches of horseshoe crabs were collected from recently laid horseshoe crab nests on four spawning beaches in mid-May 2019 by digging carefully down into the sand until a clutch was reached [44]. The eggs of each clutch adhered to each other in an exudate forming an egg-shaped or spherical mass. Although there were nearby clutches in the same 0.5 m area, each collected clutch was at least 1 m away from another. Horseshoe crab eggs were collected from the “surface” by walking into the water where eggs were concentrating, rather than right at the surf line where they would be mixed with sand and vegetation debris [22]. Samples sufficient for analysis were collected at intervals along the high tide line (or in the surf). Surface eggs are the ones available to shorebirds, gulls, and other species (e.g., fish, turtles) eating them from the surface or water. In both cases, eggs were relatively fresh because they were green. As eggs develop in clutches they lose the green color, and eggs on the surface can be green (e.g., recently laid), or become brown as they dry out. Surface eggs were collected from the surf because they were fresh and green (e.g., recently scoured up from nests). The clutches were placed into separate plastic bags for analysis at the Environmental and Occupational Health Sciences Institute of Rutgers University.

All methods were approved by the Rutgers University Institutional Animal Care and Use Committee (Protocol # 92-036) and were collected under appropriate collecting permits from the State of New Jersey (NJDEP # 2023-5002). The Rutgers IRB number reflects that the protocol was first approved in 1992 and was the 36th protocol approved that year. Subsequently, protocols are approved every three years, but retain the same original number.

### 2.2. Metals Analysis

Horseshoe crab eggs were kept frozen until they were transferred to the Elemental Analysis Laboratory of the Environmental and Occupational Health Sciences Institute at Rutgers University. Eggs of crabs are very small (144–209 eggs/mL, [22]), and were separated from sand with the use of a series of small sieves. Where necessary, tiny detritus particles were removed with a plastic tool. For each sample, 0.5 g of eggs were used (0.5 g = 250 ± 10 eggs).

Total mercury (Hg) was analyzed by Perkin-Elmer FIMS-100 mercury analyzer by cold vapor atomic absorption spectrophotometry. Other metals were analyzed using Perkin Elmer 5100 flameless graphite furnace atomic absorption. All laboratory equipment and containers were washed in 10 % HNO solution and rinsed with deionized water prior to each use. Great care was taken in the analysis to remove sand from the eggs before digestion. Eggs were digested in 70 % Ultrex ultrapure nitric acid and deionized water in a microwave. Instrument detection limits were 0.02 ppb for arsenic (As) and cadmium (Cd), 0.08 ppb for chromium(Cr), 0.15 ppb for lead (Pb), and 0.2 ppb for mercury (Hg) and selenium (Se). All specimens were analyzed in batches with known standards, calibration standards, and spiked specimens. Blanks, standard calibration curves, and spiked matrix specimens were used to monitor assay performance for all batches. Certified Reference Material (CRM) DORM-“Dogfish Muscle Certified Reference Material for Trace Elements” from the national Institute of Standards and Technology (NIST) was used for cold vapor atomic absorption spectroscopy (Hg). “Trace Metals in Natural Water” from the National Institute of Standards and Technology (NIST) was used for Zeeman graphite furnace atomic absorption spectroscopy (As, Cd, Cr, Pb and Se) quality control. All concentrations are expressed in ppb (ng/g, wet weight) for total metal. Recoveries ranged from 87% to 101%. Batches with recoveries of less than 85% were re-analyzed (although there were none in this study). The coefficient of variation on replicate, spiked samples ranged up to 10%.

We used non-parametric procedures (Kruskal Wallis test, PROC NPAR1WAY [49]) to determine species-related differences in heavy metal levels, and Kendall tau correlations for determining relationships among metal levels in the horseshoe crab egg clutches. These non-parametric tests were used because they are more conservative and are best suited for small datasets [50].

## 3. Results

### 3.1. Metals in Horseshoe Crab Eggs from Delaware Bay

Normally, samples of horseshoe eggs (or other species) are collected from one or several beaches, pooled, and analyzed, providing levels of metals in tissues of a particular species. Thus, for comparisons with the literature, metal (and metalloid) levels in all the eggs of horseshoe crabs collected from New Jersey in 2019 are given in Table 1. Levels of Cd were lowest, and levels of Se were highest.

### 3.2. Comparison of Metals in Eggs from the Surface and Clutches

There were few differences in metals levels in the horseshoe crab clutches compared to eggs found on the surface (Table 2). As was higher in clutches, and significantly lower in the surface eggs. In contrast, Cd levels were higher on surface eggs than in clutches. There were no significant differences in Cr, Pb, Hg, and Se. Thus, for these latter metals the clutches were representative of the surface eggs available to the shorebirds.

### 3.3. Locational Comparisons

There were significant geographical differences in all metal levels except for Pb (Table 3). The highest levels of As and Se were at Villas (southern-most sample), the highest levels of Cd and Cr were at Reeds, and the highest level of Hg was at Moore’s Beach. There was an order of magnitude difference in metal levels among sites only in Cr.

### 3.4. Correlations among Metals in Egg Clutches

One of the interesting aspects is whether the metals in horseshoe crab clutches are correlated. In other words, if one metal is high, are other metals in the same clutches high as well? There were some correlations, but they were not very high (Table 4). As was negatively correlated with Cd, Cr, and Pb; Cd was positively correlated with Pb and negatively correlated with Hg; Cr was positively correlated with Pb, and Hg was negatively correlated with Pb.

## 4. Discussion

The data in this paper are relevant to the following issues: (1) differences between surface and egg clutches, (2) locational differences within Delaware Bay, (3) temporal comparisons with levels from previous years, (4) correlations among metals, and lastly and most importantly, (5) the implications of the levels for crabs or their predators. Horseshoe crabs play a critical keystone role in the food web in Delaware Bay [18,51]. Each will be discussed below.

### 4.1. Contaminants in Surface Eggs versus Those in Egg Clutches

One hypothesis of this study was that there might be a difference in the levels of metals in eggs on the surface than those in clutches below the surface because the eggs on the surface are essentially scoured by the strong surf and shifting sand, whereas those buried below the sand are un-scoured and still contain the exudate from the females (that held the eggs together in a mass). Four out of six elements were not different between surface and clutch (Table 2). However, As was significantly higher and Cd was significantly lower in clutches than in surface eggs. It remains unclear why there is a difference in these two metals in surface versus clutch eggs. In both cases the eggs are relatively fresh (as they were green, and they age to a brown on the surface, and lighter in clutches). The possibility remains that the exudate (washed off by scouring) might contain additional metals as a way of excreting heavy metals by females. It is also possible that additional heavy metals adhere to the surface of the eggs that are in the broiling surf waters, but both require additional study in another year.

As the terms imply, surface eggs are on the surface and available to foraging shorebirds, whereas clutches are buried and not generally available to shorebirds. Surface eggs are brought to the surface when nests near low tide range are not buried deep enough and are scoured to the surface by the surf and bioturbation [21,28,52]. Shorebirds, including the threatened red knot, eat almost entirely the eggs of horseshoe crabs while on the Delaware Bay stopover. Previous work has indicated some differences in the levels of these same metals in the blood of species of shorebirds collected early in May versus later in May [53]. That is, shorebirds are acquiring metals while in Delaware Bay, but some species acquire more than others. Ruddy turnstones (*Arenaria interpres*) had higher levels of Cd in whole blood (mean of 5 ppb compared to <3 ppb for other shorebirds) and Pb (mean of 155 ppb vs. <90 ppb for other shorebirds) and Hg 40 ppb vs. <25 ppb for other shorebirds) in their blood than did the other species [18]. This might suggest a differential in eggs that are exposed to scouring because unlike the other species, ruddy turnstones are able to dig down to find some clutches (Burger, pers. obs). A difference in the levels in metals of surface versus clutches buried below the ground might explain some of the differences in metal levels in the blood of shorebirds. However, clutches had significantly lower levels of Cd (and similar levels of Pb and Hg) compared with surface eggs, which does not account for this difference in the blood of shorebirds.

Another possible explanation is that under some circumstances there are clutches (albeit mainly parts of clutches) on the surface when they are first churned up at the tide line. These clutches were initially discovered because gulls were concentrating in these areas, and some complete, recently exposed clutches were found. These are at the mid-tide as the tide is receding and represent clutches that have just been churned up. Indeed, Jackson et al. [52] found that nearly four times as many eggs were exhumed in the middle foreshore than in the upper reaches of the beach where crabs also lay. It may be that shorebirds sometimes have a significant proportion of eggs from clutches where the eggs have remained bound together. Recently laid eggs are green, and similarly, in recently churned up clutches the eggs are green, mixed with some grains of sand.

### 4.2. Locational Differences

There are three types of spatial differences in metal levels in horseshoe crabs: along the Jersey shore of Delaware Bay, along the Delaware side of the Bay, and along the Atlantic coast. In the present study, there were significant locational differences in metal levels, with most metals being higher in the lower two sampling sites of the NJ side of Delaware Bay (Table 3). Higher levels in the southern part of the bay are not what was expected because this part of the bay is wider and closer to the Atlantic Ocean with a regular tidal regime, and dilution should be greater given that most of the pollution is coming from metropolitan areas via the Delaware River (Figure 1). Presumably contaminants in the eggs of the crabs reflect rather recent exposure, when the females are in the bay as they spend considerable time there before spawning and are observed in the bays by the beaches for some time before spawning (Burger, unpubl.). After breeding, the crabs return to the continental shelf and may return to the bay the following spring although there is some movement in the region from the Barnegat Bay region to Virginia [26].

Spatial differences on the Delaware side of Delaware Bay were examined previously, but horseshoe crab eggs were collected on only the lower bay (although there were four sampling sites [46]). Because the sites were all relatively close together on the southern reach of Delaware Bay, there was no clear pattern in metals.

For comparison, levels of metals were examined in eggs of horseshoe crabs from nine estuaries and bays from Maine to Florida [45]. There were significant spatial differences along the Atlantic Coast for all metals, with the greatest geographical differences for As (highest in Florida), Pb (highest in Maine), and manganese and Hg (highest in Massachusetts). At the time of that coastal study [45], the levels in Delaware Bay were generally in the middle range of exposures. The samples for that study [45], however, were collected in 2000, and another Atlantic coast-wide study is warranted.

Studies from Long Island, New York, conducted in 2015, examined metals in embryos and larvae of horseshoe crabs [54]. Most metal levels increased from the embryo (egg) stage to the larvae stage. Bakker et al. [54] study found locational differences in the metal levels, but they were not related to levels in water or sediment, suggesting that abiotic factors and uptake pathways account for this lack of differences [54,55]. At all sampling sites in the Bakker et al. studies, As levels were higher than all other metals [48,55]; this was true in this study as well.

### 4.3. Temporal Differences in Metal Levels

Metal levels in the eggs of horseshoe crabs have been measured since the early 1990s in Delaware Bay [43,44]. Burger and Tsipoura [44] found the following, adding the 2019 data reported in this paper:

(1) As (measured only since 1999) was less than 3000 ppb, then increased in the 4000 ppb to 6000 ppb range until 2012, and in 2019 it was 5247 ppb.
(2) Cd usually averaged below 10 ppb and has remained low in most years.
(3) Cr was quite high in the early 1990s (averaged about 5000 ppb), but declined to about 2050 ppb in 1995, and stayed below an average of 100 ppb since the late 1990s.
(4) Pb averaged nearly 550 ppb in 1994, declined to just over 200 ppb in the next year, and has remained low.
(5) Hg was variable in the 1990s (average of 22 ppb to 300 ppb) and decreased to below 20 ppb thereafter.
(6) Se was 2000–4000 ppb in the mid-1990s, then went down to around 1000 ppb in the late 1990s to 2012 in some sites on the Bay. It was 1726 ppb in 2019.

These temporal data indicate that horseshoe crab egg levels of Cr, Pb, Hg, and Se have declined since the 1990s, As has increased, and Cd has remained low with some variations. It is not clear why As is increasing, nor why Hg varies more than the other metals. However, the spatial data (within Delaware Bay [46]), and along the Atlantic coast [45]) and the temporal data (1993–2019 [44]) make horseshoe crab eggs an excellent bioindicator of metal contamination, and potential exposure for their predators.

### 4.4. Correlations among Clutches

There were some significant correlations among metals for clutches (refer to Table 4). As, relatively high in the eggs of horseshoe crabs, was negatively correlated with Cd, Cr, and Pb. Further, Pb was positively correlated with Cd and Cr and negatively correlated with Hg. These relationships deserve more examination because in general, one might expect metals to be correlated because the eggs were collected in different locations, and eggs from females that spawned farther north (e.g., closer to the source of pollution) might be exposed to above levels of contaminants generally. However, the eggs collected from farther south in the bay generally had higher levels than those from norther beaches in the bay.

### 4.5. Potential Effects of Contaminants in Horseshoe Eggs

#### 4.5.1. Effects on Horseshoe Crabs

Contaminant levels in horseshoe eggs are important for two reasons: (1) they are indicative of female crab toxic exposure, and (2) they are indicative of exposure of species that eat the eggs. Horseshoe crabs have declined in many places along the Atlantic coast [24,25] and in Asia [56]. While the decline of the Atlantic horseshoe crab was largely the result of increased take for bait, the decline of the Japanese horseshoe crab was partly due to high levels of coastal pollution [57]. This suggests that the potential effect of other stressors, such as heavy metals, needs to be continually examined in the Atlantic horseshoe crab, as pollution from increasing urbanization may increase the threat.

It is difficult to examine the effects of metals in field situations, except by (1) exposing animals in the field and observing effects, (2) examining animals in the wild for differences in metal exposure and subsequently observing morphological or behavioral effects, or (3) finding animals with morphological, behavior, or other effects and subsequently measuring metal levels in their tissues and correlating exposure with effects. All of these are labor intensive, challenging, and hard to conduct in the field. Thus, laboratory experiments can provide an indication of potential effects. The difficulty is that the link between exposure and effects (established in laboratory studies), is not connected to tissue levels of those effects. That is, the laboratory experiments provide exposure levels and show effects, but do not normally connect them by measuring the levels of metals in the organ or animals at the time effects are observed in the laboratory.

Laboratory experiments with Atlantic horseshoe crab larvae indicate some mortality and developmental effects when exposed to heavy metals [58,59], but they did not examine all the metals examined in the present study. In their experiments they found different effects on survival, molting, and regeneration that differed by metals, with the greatest effects associated with Hg, followed by Cd, Cr, and then copper. Unexpectedly, they found that larvae from a more polluted bay (Sandy Hook, NJ, USA) were more susceptible to metals than those from the less polluted bay (Delaware Bay [59]. Itow et al. [60] also found that metals inhibited limb regeneration. Botton et al. [59] commented that horseshoe crab larvae are more resistant to metal exposure compared to other marine crustacea [59], perhaps because of the horseshoe crab’s 430-million-year lineage [61].

While these experiments are very important, they did not measure the amount of each metal or metalloid in the crabs themselves when they found an effect. The relationship between contaminants in eggs and the tissues of horseshoe crabs is critical (not just the exposure levels in water). There is clearly maternal exposure because unlaid crab eggs contain metals, which they obtained from their mother; and levels of metals in legs of females are correlated with metals in their eggs for Hg, Se, Pb, and Hg [55]. Understanding the relationship between exposure levels, effects, and tissue levels in exposed crabs is a critical step. The lack of data makes it difficult to determine the effects of metal levels from field concentrations. The metal loading in Delaware Bay comes from industrialized Philadelphia [62,63], but metal levels should be lower in the lower Bay because of dilution and daily tidal flow from the Atlantic Ocean (refer to Figure 1).

Similar concerns are being expressed for horseshoe crabs in Asia. For example, studies of tri-spine horseshoe crab (*Tachypleus tridentatus*) demonstrate that they accumulate metals as well [64,65]. They found the highest levels in gills, tissues that have not been examined in the Atlantic horseshoe crabs, and that there were effects on antioxidant parameters, especially during the recovery periods [65]. In laboratory experiments they found that the tri-spine horseshoe crab (as well as the Atlantic horseshoe crab exposed similarly) had developmental abnormalities that were caused by exposure to metals. The severity in effects was as follows (from greatest to least): Hg, Cr and Cd, and Pb, of the metals examined [57]. Similarly, Kwan et al. [66,67] reported effects on growth and hemolymph quality in tri-spine horseshoe crabs exposed to metals, making it a potentially useful bioindicator of effects [66,67].

#### 4.5.2. Effects on Shorebirds

**Figure 2:**
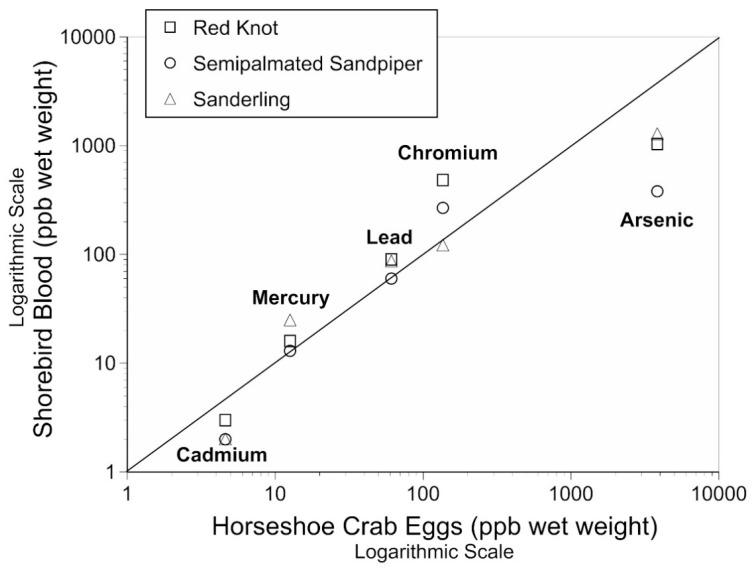
Relationship of mean levels of metals and metalloids in the blood of three species of shorebirds trapped on their Delaware Bay stopover to the levels of metals in Delaware Bay horseshoe crab eggs (both collected in Delaware Bay in 2012; Figure 2 from Burger et al. [47], Toxics 2017).

The second reason for examining metals and metalloids in horseshoe crab eggs is their role in the food web of Delaware Bay [18]. As mentioned above, horseshoe crabs are the primary prey for migrant shorebirds in May each year [29,30,31,32], and red knot’s survival is linked to horseshoe crab spawning abundance [68,69]. Therefore, contaminants in eggs of horseshoe crabs are critical because there is a direct relationship between the metals in crab eggs and the levels in the blood of shorebirds [53]. The tight relationship between levels in metals in crab eggs and those in the blood of shorebirds reported previously [47] corroborates that the shorebirds are mainly eating the eggs of horseshoe crabs while on the Delaware Bay stopover now, as they were in the past [23]. The relationship of shorebird blood and horseshoe crab blood is illustrated in Figure 2 for clarity of the relationship (Figure 2 from Burger et al. [47]). Shorebirds migrating through Delaware Bay are mainly eating horseshoe crab eggs making it possible to examine the relationship between horseshoe crab eggs (prey) and levels of metals in the blood of predators (shorebirds). The levels of most metals in shorebirds were like those in crab eggs, but both Cd and As were lower. That study did not examine levels of Se, the metal with the highest levels, nor did it examine turnstones [23]. These relationships need to be examined in another year to determine its consistency, and if As and Cd levels in shorebird blood are lower than in horseshoe crab eggs, suggesting that these metals are not absorbed by some species of shorebirds as much as the other metals.

One anomaly is worth noting—As was much higher in clutches than in surface eggs, but As was lower than expected in bird blood (Figure 2). As exhibits complex geochemistry in the abiotic and biotic compartments of the estuarine environment [70]. It exists as organic arsenosugars, as inorganic solutes, and adsorbed to suspended particular matter. It is not safe to assume that the As found in the present study represents a “contaminant” since As occurs naturally in sea water. Although dissolved arsenate predominates in open water, suspended particulate occurs right along the shore [70] frequented by horseshoe crabs. One explanation for the findings may be the incorporation of particulate As in the exudate when the clutch is deposited, versus the subsequent washing away of particulate from the surface eggs in the water column.

There are no comparable studies examining the levels of metals in the blood of other birds (e.g., gulls feeding on the eggs), and other marine species (young fish and hatchling turtles) that eat horseshoe crabs as well. This would be a fruitful avenue of future research, as these species eat other prey as well, while during their stopover the shorebirds eat only horseshoe crab eggs [23].

Finally, the tight relationship between metal levels in the blood of shorebirds and horseshoe crab eggs also raises the possibility that any accident or spill of chemicals or oil into the Delaware Bay waters could conceivably be concentrated in horseshoe crabs and their eggs, and subsequently expose migrant shorebirds at a time when they are foraging on one type of prey.

## 5. Conclusions

Determining metal levels in the eggs of horseshoe crabs provides information on the risk to the developing embryos of crabs, maternal levels, and the risk to species that prey upon them. Several laboratory studies have demonstrated developmental effects of several metals, particularly Hg, and that metal pollution is one of the main causes of horseshoe crab declines in Asia. There were few differences in metal levels between surface eggs and clutches below the surface, although clutches had significantly higher levels of As. However, there were some locational differences, with levels being highest at the southern sampling areas of Delaware Bay. This was surprising, since these locations are farther from the source of pollution (Philadelphia and other industries in the Bay). Laboratory experiments and field studies have shown that metals, particularly Hg, can adversely affect the development and behavior of horseshoe crabs. Horseshoe crabs are a keystone species in Delaware Bay. Many species eat the eggs of horseshoe crabs, including the threatened red knot and other species of shorebirds that are declining, as well as young fish and young turtles. Since there is a direct correlation between the metals in horseshoe eggs and in the blood of shorebirds, metals in crab eggs should continue to be monitored as a potential early warning of any future effects.

## Figures and Tables

**Table 1:** Metal levels (ppb, wet weight) (ng/g) in eggs of horseshoe crabs from Delaware Bay, 2019. All eggs were collected on the New Jersey side of Delaware Bay (includes surface eggs and eggs from clutches, n = 50 samples).

Metal	Mean + Standard Error	Range (Minimum–Maximum)
As	4529 ± 484.3	1400–18,000
Cd	9 ± 2.1	0.07–64
Cr	102.1 ± 11.1	21–390
Pb	114.8 ± 19.2	7.9–570
Hg	12.7 ± 1.0	0.1–44.8
Se	1688 ± 169.7	410–4700

**Table 2:** Metal levels (mean ± SE, ppb, wet weight) (ng/g) in horseshoe crab clutches from Reeds Beach compared with surface eggs collected at Reeds Beach. Surface eggs, the food of the shorebirds, were collected from the surf zone in 2019. Kruskal–Wallis Chi Square values and *p* values compare metal levels between clutches and surface eggs. NS = not significant.

Metals	Clutches (Reeds)	Surface (Reeds)	X^2^ (*p*)
Sample size	16	12	
As	3375 ± 254	2058 ± 134	11.5 (0.0007)
Cd	6.9 ± 1.8	34.3 ± 8.1	11.4 (0.0007)
Cr	133 ± 21.8	111 ± 24.4	1.5 (NS)
Pb	136 ± 18.6	355 ± 148	0.86 (NS)
Hg	11.1 ± 1.5	10.3 ± 2	0.05 (NS)
Se	1667 ± 277.2	1837 ± 429	0.29 (NS)

**Table 3:** Levels of metals (±standard errors, ppb, ng/g, wet weight) differences in horseshoe crab clutches in several sites in New Jersey in 2019. Kruskal–Wallis Chi Square values compare levels among sites. Sites listed from south to north.

Metals	Villas	Reeds	Moore’s	Fortescue	X^2^ (*p*)
Sample	6	16	10	6	
As	11,117 ± 1962	3375 ± 254	5110 ± 614	4600 ± 1024	6.48 (0.09)
Cd	4.39 ± 3.3	6.9 ± 1.7	6.4 ± 3.2	1.1 ± 0.16	13.2 (0.004)
Cr	40.3 ± 7.6	133.4 ± 21.8	85.4 ± 20.3	96.5 ± 19.3	14.5 (0.002)
Pb	64.2 ± 27.8	135.6 ± 18.6	52 ± 13.4	29 ± 13.5	5.08 (NS)
Hg	12.3 ± 1	11.1 ± 1.48	17.7 ± 3.3	12 ± 1	14.7 (0.002)
Se	2968 ± 624	1667 ± 277	785 ± 62	2200 ± 313	15.1 (0.002)

**Table 4:** Kendall tau correlations among metal levels in clutches of horseshoe crab eggs from Delaware Bay (N = 34, * = *p* < 0.05, ** = *p* < 0.01).

	As	Cd	Cr	Pb	Hg	Se
As	--					
Cd	−0.25 *	--				
Cr	−0.44 **	0.17	--			
Pb	−0.27 *	0.39 **	0.34 **	--		
Hg	0.2	−0.36 **	−0.09	−0.31 **	--	
Se	0.09	−0.22	−0.08	0.03	0.005	--

## Data Availability

The data are available from the author upon request.
